# Necessity of energy layer repainting for proton radiotherapy in breast cancer patients

**DOI:** 10.1016/j.phro.2026.100971

**Published:** 2026-04-17

**Authors:** Bojan Štrbac, Truls Andersen, Jeffrey Free, Pietro Piscotta, John Maduro, Johannes A. Langendijk

**Affiliations:** Department of Radiation Oncology, University Medical Center Groningen, University of Groningen, Groningen, the Netherlands

**Keywords:** Proton therapy, Pencil beam scanning, Breast cancer, Respiratory motion, Interplay effect, Repainting

## Abstract

This study assessed the necessity of energy layer repainting for free-breathing breast proton therapy using pencil beam scanning (PBS). Ten patients were analyzed via log file-based 4D dose reconstruction across three scenarios: no repainting (1×), 3×, and 5× energy-layer repainting. Median target coverage deviations (D_98_%) remained within ±4.1% for all strategies, with no statistically significant differences (p > 0.05). Respiratory motion was minimal (mean <2.2 mm)**,** while delivery time increased substantially with repainting. For free-breathing breast PBS with minimal respiratory motion, small target excursions, intra-fraction delivery averaging, and conventional fractionation rendered energy layer repainting unnecessary.

## Highlights

• Layer repainting did not improve target coverage for breast proton therapy.
• Respiratory motion for breast and nodal targets was minimal (mean <2.2 mm).
• Target dose coverage deviations were within ±4.1% for all scenarios.
• Beam-on time increased from 2.5 to 6.5 min with 5× repainting.
• Small motion and fractionation averaging preserved coverage without repainting.

## Graphical abstract

**Figure:**
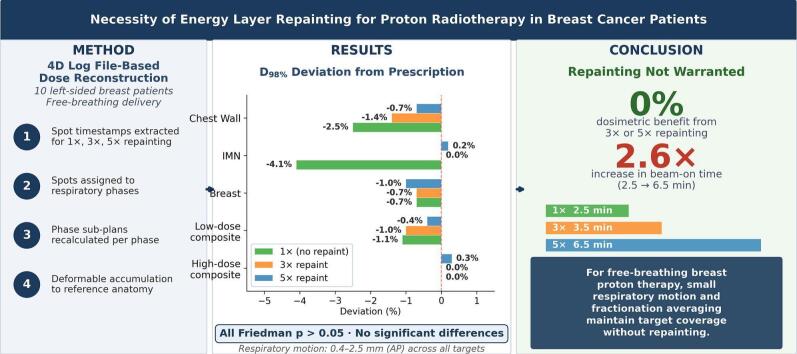

## 1 Introduction

Proton radiotherapy is increasingly considered for selected breast cancer patients primarily due to its ability to reduce integral cardiac and pulmonary dose compared with conventional photon radiotherapy [1], [2]. Specifically, for left-sided breast cancer, this benefit significantly lowers the risk of long-term cardiac toxicity [3]. However, as proton radiotherapy is not the standard of care, its use requires careful consideration of both its superior dose distribution and its inherent technical challenges related to motion management.

Among available proton delivery techniques, pencil beam scanning (PBS) allows for highly conformal dose delivery but is inherently sensitive to intra-fractional respiratory motion. The resulting interplay effect, the dynamic synchronization of the moving anatomy and the sequential spot scanning, may theoretically cause under-dosage (cold spots) in the clinical target volume (CTV) or unintended hot spots in nearby organs at risk (OARs) [4].

Several motion-management strategies exist for treatments. In photon breast radiotherapy, deep-inspiration breath-hold (DIBH) is a widely adopted technique to reduce both motion and cardiac dose. In proton breast treatments, DIBH may be used, but free-breathing (FB) delivery is frequently employed when motion is small and conventional fractionation provides sufficient averaging of residual interplay effects [5], [6].

Repainting is a delivery technique designed to mitigate the interplay effect by delivering each energy layer (layer repainting) or discrete portions of the target volume (volumetric repainting) multiple times. While volumetric repainting offers theoretical robustness, layer repainting is more commonly implemented due to hardware constraints. By repeating the scanning sequence, motion-induced dose heterogeneities tend to average out over the total delivery time. In high-motion sites such as mobile lung tumors, layer or spot-adapted repainting is often essential to maintain dose coverage [7], [8].

In contrast, breast and regional nodal targets typically show much smaller respiratory excursions than high-motion tumors. Reported displacements of the breast and chest wall are generally on the order of a few millimeters, predominantly in the anterior–posterior (AP) direction [6], [10]. It remains unclear whether layered repainting is necessary to ensure adequate target coverage and homogeneity in breast PBS, given that inherently small respiratory excursions and conventional fractionation may suffice without added complexity. Layer repainting, although less complex than volumetric repainting, still increases delivery complexity and significantly prolongs beam-on time [9].

In this investigation, we used a log file-based dose reconstruction and accumulation method to quantitatively assess the necessity of layered energy repainting in free-breathing left-sided breast cancer patients. The primary aim was to determine if layered repainting improved CTV coverage or homogeneity sufficiently to justify its added delivery time and complexity.

## 2 Materials and methods

### 2.1 Patient selection and imaging

Ten left-sided breast cancer patients previously treated with proton therapy were selected. This study was conducted under the ethical approval of the Standard Follow-up Program Mamma (SFP MAMMA; approval no. 2017/509; ClinicalTrials.gov: NCT06429995). All patients had indications for comprehensive irradiation including internal mammary lymph nodes (IMNs). Each patient underwent a free-breathing (FB) planning computed tomography (CT) and a four-dimensional CT (4DCT) for motion assessment. The 4DCT consisted of ten respiratory phase images reconstructed using a respiratory gating belt (AZ-733VI, Anzai Medical Co). The average CT scan served as the reference for nominal planning and delineation of CTV structures (breast/chest wall, regional nodes), which were subsequently propagated to all 4DCT phases using deformable image registration (DIR; ANACONDA, RaySearch Laboratories, Stockholm, Sweden). Informed consent was obtained for the use of patient data.

### 2.2 Treatment planning

Proton PBS plans were generated in RayStation version 2023B (RaySearch Laboratories) for each patient, typically utilizing two or three beams. All plans were delivered in 15 fractions. Prescriptions were patient-specific and followed a simultaneous integrated boost (SIB) scheme when a boost volume was indicated: the low-dose composite CTV (CTV_low), encompassing the elective volume including the breast/chest wall and regional nodes, received 40.05–44.66 Gy(RBE), while the high-dose composite CTV (CTV_high), encompassing the tumour bed boost volume, received 53.40–58.74 Gy(RBE). A subset of patients without an indicated boost received a single uniform prescription of 40.05 Gy(RBE) to the comprehensive target.

A constant relative biological effectiveness (RBE) value of 1.1 was uniformly assumed for all dose calculations, and all reported doses are expressed in Gy(RBE).^1^ All plans were robustly optimized on the average CT using a comprehensive planning parameter set that included a ±5 mm setup uncertainty (in all three translational directions) and a ±3% proton range uncertainty. Layer repainting with a nominal factor of 5 was enabled in the planning module, instructing the machine to deliver each energy layer five times, which affects only the temporal delivery pattern; the robust optimization itself addresses geometric setup and range uncertainties but does not account for interplay effects.

### 2.3 Repainting scenarios and log file modification

Three delivery scenarios were analyzed retrospectively per patient: 1× (no repainting, single pass per layer), 3×, and 5 × energy-layer repainting. All three scenarios were built upon the same fundamental plan (identical spot positions and monitor units). Delivery log files were retrieved and modified to simulate the scenarios by systematically removing or truncating the repeated passes of each energy layer.

### 2.4 4D dose reconstruction and accumulation

A log file–driven dose reconstruction methodology [11], [12] was implemented. For each patient and repainting scenario, the delivery log was synchronized with one of three representative breathing traces (shallow, medium, or deep) to model motion dynamics. Each delivered spot was assigned to one of the ten phases based on the instantaneous phase of the breathing trace at its timestamp. Each resulting phase-specific sub-plan was recalculated on the corresponding phase image. Finally, all ten phase doses were deformably registered to the static end-exhale (50%) reference anatomy using deformation vector fields (DVFs) and algebraically summed to obtain the final 4D accumulated dose for each scenario.

### 2.5 DVF-based motion analysis

The DVFs calculated between the end-inhalation and end-exhalation CT phases were analyzed to quantify the respiratory motion of the target volumes. Statistics included the mean displacement in the right–left (RL), AP, and superior–inferior (SI) directions, as well as the mean and maximum vector magnitude of motion. Patient-level group summaries were generated for each anatomical group and averaged across the cohort.

### 2.6 Dose endpoints and statistics

The primary dose-volume metrics were D_98%_ (target coverage) and D_2%_ (hotspot metric). Values derived from the accumulated dose were expressed as the percentage dose deviation (PD) from the prescribed dose. Non-parametric paired tests were employed: a Friedman test assessed overall differences, and subsequent pairwise Wilcoxon signed-rank tests (denoted 5v1 for 5 × versus 1×, 3v1 for 3× versus 1×, and 5v3 for 5× versus 3×) were conducted with a Bonferroni correction (adjusted alpha = 0.017).

## 3 Results

**Fig. 1:**
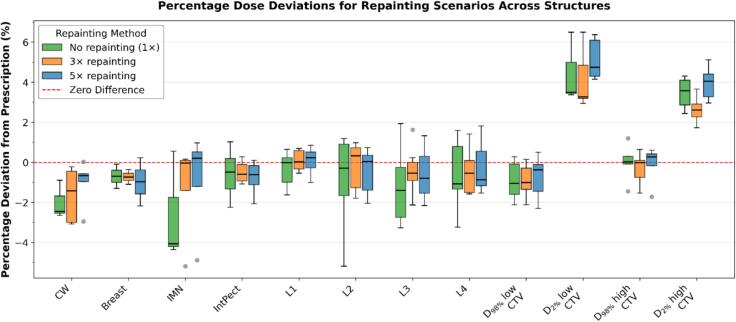
Boxplots of percentage dose deviations from prescription (PD) for D_98%_ and D_2%_ across structures and repainting scenarios (1×, 3×, 5×). The horizontal line indicates zero deviation.

No clinically relevant or statistically significant differences in target coverage or dose homogeneity were observed between 1×, 3×, and 5× repainting. For all CTV structures, Friedman tests yielded p > 0.05, and Kendall’s W values were generally below 0.3. Median percentage deviations of D_98%_ from prescription were within approximately ±4.1% for all structures and repainting scenarios. For the chest wall CTV, PD(D_98%_) medians [IQR] were −2.5% [−2.6, −1.7] for 1×, −1.4% [−3.0, −0.4] for 3×, and −0.7% [−1.0, −0.6] for 5×. For the IMN CTV, PD(D_98%_) medians [IQR] were −4.1% [−4.2, −1.7] for 1×, 0.0% [−1.4, 0.1] for 3×, and 0.2% [−1.2, 0.5] for 5×. The low-dose composite CTV showed PD(D_98%_) medians [IQR] of −1.1% [−1.6, −0.1] for 1×, −1.0% [−1.3, −0.3] for 3×, and −0.4% [−1.4, −0.1] for 5× (Table 1, Fig. 1).

**Table 1:** Median percentage dose deviations from prescription (PD) with interquartile ranges [IQR] for D_98_% and D_2_% by structure and repainting scenario (1×, 3×, 5×). Friedman p-values, Kendall's W, and pairwise Wilcoxon signed-rank test results are reported. Pairwise comparisons are denoted 5v1 (5× versus 1×), 3v1 (3× versus 1×), and 5v3 (5× versus 3×). Adjusted p-values use Bonferroni correction (α = 0.017). CW = chest wall; IMN = internal mammary nodes; IntPect = interpectoral nodes; L1–L4 = axillary levels 1–4; low CTV = low-dose composite CTV; high CTV = high-dose composite CTV.

Structure/Metric	PD 1 × [%] med [IQR]	PD 3 × [%] med [IQR]	PD 5 × [%] med [IQR]	Friedman p	W	Wilcoxon p (5v1, 3v1, 5v3)	Adjusted p (5v1, 3v1, 5v3)
CW (D_98%_)	−2.5 [−2.6, −1.7]	−1.4 [−3.0, −0.4]	−0.7 [−1.0, −0.6]	0.10	0.78	1.00, 0.25, 0.19	1.00, 0.75, 0.56
Breast (D_98%_)	−0.7 [−1.0, −0.4]	−0.7 [−0.9, −0.5]	−1.0 [−1.6, −0.4]	1.00	0.00	1.00, 1.00, 1.00	1.00, 1.00, 1.00
IMN (D_98%_)	−4.1 [−4.2, −1.7]	0.0 [−1.4, 0.1]	0.2 [−1.2, 0.5]	0.67	0.12	1.00, 1.00, 0.25	1.00, 1.00, 0.75
IntPect (D_98%_)	−0.5 [−1.3, 0.2]	−0.6 [−0.9, −0.1]	−0.6 [−1.1, −0.2]	0.34	0.20	1.00, 1.00, 0.13	1.00, 1.00, 0.39
L1 (D_98%_)	0.0 [−1.0, 0.2]	0.0 [−0.3, 0.6]	0.2 [−0.3, 0.5]	0.61	0.17	0.22, 1.00, 0.47	0.66, 1.00, 1.00
L2 (D_98%_)	−0.3 [−1.7, 0.9]	0.3 [−1.3, 0.7]	0.0 [−1.4, 0.3]	0.51	0.22	1.00, 1.00, 0.16	1.00, 1.00, 0.47
L3 (D_98%_)	−1.4 [−2.7, −0.2]	−0.5 [−0.9, 0.0]	−0.8 [−1.5, 0.3]	0.51	0.22	1.00, 1.00, 1.00	1.00, 1.00, 1.00
L4 (D_98%_)	−1.1 [−1.3, 0.8]	−0.5 [−1.5, 0.1]	−0.9 [−1.2, 0.6]	0.31	0.23	0.31, 1.00, 0.16	0.94, 1.00, 1.00
D_98%_ low CTV	−1.1 [−1.6, −0.1]	−1.0 [−1.3, −0.3]	−0.4 [−1.4, −0.1]	0.29	0.25	1.00, 0.24, 1.00	1.00, 0.71, 1.00
D_2%_ low CTV	3.5 [3.4, 5.0]	3.3 [3.2, 4.8]	4.7 [4.3, 6.1]	0.53	0.15	0.33, 0.18, 0.13	1.00, 0.54, 0.38
D_98%_ high CTV	0.0 [−0.1, 0.3]	0.0 [−0.7, 0.1]	0.3 [−0.2, 0.4]	0.55	0.12	1.00, 0.13, 1.00	1.00, 0.38, 1.00
D_2%_ high CTV	3.6 [2.9, 4.1]	2.6 [2.3, 2.9]	4.0 [3.3, 4.4]	0.02	0.84	0.13, 0.06, 0.06	0.38, 0.19, 0.19

For D_2%_, differences between repainting scenarios were likewise small. Only the D_2%_ of the high-dose CTV showed a significant Friedman result (p = 0.02); however, none of the post-hoc Wilcoxon signed-rank comparisons remained significant after Bonferroni correction (all adjusted p > 0.05).

The DVF analysis confirmed that all targets in this cohort exhibited only minimal respiratory motion. The mean vector magnitude of motion across nodal and breast CTVs ranged from 0.9 to 1.6 mm. The thoracic wall CTV exhibited the largest motion with a mean magnitude of 2.2 mm, consistent with rib-cage expansion. In all groups, motion was greatest in the AP direction and well within the ±5 mm planning margin. The maximum individual voxel displacement within any CTV reached 3 mm, observed at the anterior border of the chest wall CTV.

Layered repainting substantially increased the beam-on time. For 1 × delivery, a single field delivery required on average 1.2 min. With 3× repainting, the mean delivery time increased to 2.0 min. For 5× repainting, the mean delivery time was 3.2 min, with the longest beam delivery requiring up to 6 min. For a typical two-field treatment, the total beam-on time increased from approximately 2.5 min (1×) to 6–7 min (5×).

## 4 Discussion

The key finding was that for free-breathing left-sided breast patients treated with robust optimization, layered energy repainting did not provide a measurable improvement in target coverage (D_98%_) or hotspot metrics (D_2%_). Conversely, the use of repainting incurred a significant penalty, more than doubling the treatment beam-on time.

The observed lack of benefit in dose metrics from repainting was strongly supported by the DVF analysis, which confirmed consistently minimal target motion. These small, sub-centimeter displacements resulted in negligible interplay effects, which were further mitigated by intra-fraction delivery averaging over multiple respiratory cycles and the conventional 15-fraction schedule [11], [12]. It is important to note that the robust optimization employed in this study addressed geometric setup and range uncertainties but did not model or mitigate the interplay effect; the interplay was independently assessed through our 4D log file-based reconstruction.

These findings were consistent with the broader literature on motion in proton breast therapy. Flejmer et al. reported that residual free-breathing motion in PBS breast plans remained within clinically tolerable limits for typical breast geometries [5], [6], and earlier 4DCT analyses found maximum centroid displacements of 1–4 mm for the ipsilateral breast [10]. By contrast, lung interplay studies driving repainting adoption involved motion amplitudes (3–30 mm) an order of magnitude larger than ours, where benefits remained dependent on spot size and geometry [7], [8]. Our cohort sat firmly in the low-motion regime, with maximum voxel displacement below 3 mm across all CTVs. The per-patient 4D log file-based reconstruction extended these prior observations by quantifying the insensitivity of D_98%_ and D_2%_ to the repainting factor in a clinically realistic cohort with comprehensive nodal irradiation including IMNs.

In a clinical setting, the time penalty was a crucial factor. Longer beam-on times also increased the patient's discomfort and the potential for baseline drift or small patient movements, which could inadvertently degrade the dose distribution in FB treatments [11], [12]. Given the absence of advantages in dose metrics, this time penalty was not clinically justified.

A limitation of our study was that OAR doses were not separately analyzed in the 4D accumulation. However, in all patients the mean heart dose remained below 0.5 Gy(RBE), well under 2% of any of the per-patient elective prescriptions used in this cohort. Since the interplay-induced dose variations at the target level were not statistically or clinically significant, any repainting-related variation at the low-dose OAR level was expected to fall below the sensitivity of the dose calculation algorithm and to be clinically negligible. This assumption should be confirmed by future work for indications where OAR doses approach tolerance levels. Furthermore, we used representative breathing traces (shallow, medium, deep) to drive the reconstructions. While these traces were chosen to encompass the expected range of patient variability, patient-specific traces could potentially model extreme breathing irregularities more accurately.

In conclusion, for free-breathing left-breast PBS with minimal respiratory excursions and conventional fractionation, layered energy repainting did not provide a clinically relevant improvement in target dose coverage or homogeneity.

## CRediT authorship contribution statement

**Bojan Štrbac:** Writing – original draft, Visualization, Validation, Software, Project administration, Methodology, Investigation, Funding acquisition, Formal analysis, Data curation, Conceptualization. **Truls Andersen:** Writing – review & editing, Validation, Project administration, Investigation, Formal analysis, Data curation, Conceptualization. **Jeffrey Free:** Writing – review & editing, Validation, Methodology, Investigation. **Pietro Piscotta:** Writing – review & editing, Investigation, Formal analysis. **John Maduro:** Writing – review & editing. **Johannes A. Langendijk:** Writing – review & editing.

## Declaration of competing interest

The authors declare that they have no known competing financial interests or personal relationships that could have appeared to influence the work reported in this paper.

## Footnotes

1 Gy(RBE) is used according to the ICRU 93 convention for biologically weighted proton dose and is not an SI unit.

## References

[1] Mutter R.W., Choi J.I., Jimenez R.B., Kirova Y.M., Fagundes M., Haffty B.G. (2021). Proton therapy for breast cancer: a consensus statement from the Particle Therapy Cooperative Group Breast Cancer Subcommittee. Int J Radiat Oncol Biol Phys.

[2] Darby S.C., Ewertz M., McGale P., Bennet A.M., Blom-Goldman U., Brønnum D. (2013). Risk of ischemic heart disease in women after radiotherapy for breast cancer. N Engl J Med.

[3] Bradley J.A., Dagan R., Ho M.W., Rutenberg M., Morris C.G., Li Z. (2016). Initial report of a prospective dosimetric and clinical feasibility trial demonstrates the potential of protons to increase the therapeutic ratio in breast cancer compared with photons. Int J Radiat Oncol Biol Phys.

[4] Bert C., Durante M. (2011). Motion in radiotherapy: particle therapy. Phys Med Biol.

[5] Flejmer A.M., Edvardsson A., Dohlmar F., Josefsson D., Nilsson M., Witt Nyström P. (2016). Respiratory gating for proton beam scanning versus photon 3D-CRT for breast cancer radiotherapy. Acta Oncol.

[6] Flejmer A.M., Chehrazi B., Josefsson D., Toma-Dasu I., Dasu A. (2017). Impact of physiological breathing motion for breast cancer radiotherapy with proton beam scanning – an in silico study. Phys Med.

[7] Grassberger C., Dowdell S., Lomax A., Sharp G., Shackleford J., Choi N. (2013). Motion interplay as a function of patient parameters and spot size in spot scanning proton therapy for lung cancer. Int J Radiat Oncol Biol Phys.

[8] Dowdell S., Grassberger C., Sharp G.C., Paganetti H. (2013). Interplay effects in proton scanning for lung: a 4D Monte Carlo study assessing the impact of tumor and beam delivery parameters. Phys Med Biol.

[9] Zenklusen S.M., Pedroni E., Meer D. (2010). A study on repainting strategies for treating moderately moving targets with proton pencil beam scanning at the new Gantry 2 at PSI. Phys Med Biol.

[10] Qi X.S., White J., Rabinovitch R., Merrell K., Sood A., Bauer A. (2010). Respiratory organ motion and dosimetric impact on breast and nodal irradiation. Int J Radiat Oncol Biol Phys.

[11] Meijers A., Jakobi A., Stützer K., Guterres Marmitt G., Both S., Langendijk J.A. (2019). Log file-based dose reconstruction and accumulation for 4D adaptive pencil beam scanned proton therapy in a clinical treatment planning system: implementation and proof-of-concept. Med Phys.

[12] Meijers A., Seller Oria C., Free J., Bondesson D., Rabe M., Parodi K. (2020). Evaluation of interplay and organ motion effects by means of 4D dose reconstruction and accumulation. Radiother Oncol.

